# 2,6-Bis(2-hydroxy­ethyl)-8b,8c-diphenyl­perhydro-2,3a,4a,6,7a,8a-hexa­azacyclo­penta­[*def*]fluorene-4,8-dithione

**DOI:** 10.1107/S1600536809019230

**Published:** 2009-05-29

**Authors:** Zihua Wang, Hailing Xi

**Affiliations:** a Key Laboratory of Pesticide and Chemical Biology of the Ministry of Education, College of Chemistry, Central China Normal University, Wuhan 430079, People’s Republic of China; b 6th Department, Research Institute of Chemical Defence, Beijing 102205, People’s Republic of China

## Abstract

In the title mol­ecule, C_24_H_28_N_6_O_2_S_2_, the dihedral angle between the aromatic ring planes is 42.2 (1)°. In the crystal structure, the hydr­oxy groups are involved in O—H⋯S hydrogen bonding, which links the mol­ecules into corrugated layers propagating parallel to the *bc* plane.

## Related literature

For the preparation of the title compound, see: Li *et al.* (2006 ▶); Broan *et al.* (1989 ▶). For general background regarding glycol­uril and its derivatives, see Gao *et al.* (2009 ▶).
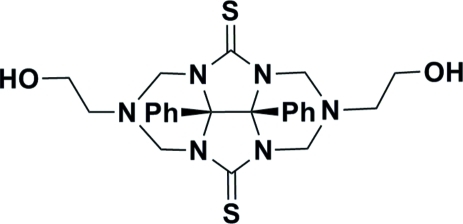


## Experimental

### 

#### Crystal data

C_24_H_28_N_6_O_2_S_2_
*M* *_r_* = 496.64
Monoclinic, 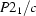

*a* = 10.8207 (3) Å
*b* = 11.9259 (3) Å
*c* = 18.7222 (5) Å
β = 95.917 (1)°
*V* = 2403.16 (11) Å^3^
*Z* = 4
Mo *K*α radiation
μ = 0.26 mm^−1^
*T* = 295 K
0.30 × 0.20 × 0.10 mm

#### Data collection

Bruker SMART APEX CCD area-detector diffractometer
Absorption correction: multi-scan (*SADABS*; Sheldrick,1996 ▶) *T*_min_ = 0.927, *T*_max_ = 0.975
26638 measured reflections
5248 independent reflections
3908 reflections with *I* > 2σ(*I*)
*R*_int_ = 0.048

#### Refinement

*R*[*F* ^2^ > 2σ(*F* ^2^)] = 0.049
*wR*(*F* ^2^) = 0.141
*S* = 1.04
5248 reflections
313 parameters
2 restraints
H atoms treated by a mixture of independent and constrained refinement
Δρ_max_ = 0.43 e Å^−3^
Δρ_min_ = −0.31 e Å^−3^

### 

Data collection: *SMART* (Bruker, 2001 ▶); cell refinement: *SAINT* (Bruker, 2001 ▶); data reduction: *SAINT*; program(s) used to solve structure: *SHELXS97* (Sheldrick, 2008 ▶); program(s) used to refine structure: *SHELXL97* (Sheldrick, 2008 ▶); molecular graphics: *SHELXTL* (Sheldrick, 2008 ▶); software used to prepare material for publication: *SHELXTL*.

## Supplementary Material

Crystal structure: contains datablocks global, I. DOI: 10.1107/S1600536809019230/cv2558sup1.cif

Structure factors: contains datablocks I. DOI: 10.1107/S1600536809019230/cv2558Isup2.hkl

Additional supplementary materials:  crystallographic information; 3D view; checkCIF report

## Figures and Tables

**Table 1:** Hydrogen-bond geometry (Å, °)

*D*—H⋯*A*	*D*—H	H⋯*A*	*D*⋯*A*	*D*—H⋯*A*
O1—H1⋯S2^i^	0.81 (2)	2.605 (19)	3.337 (2)	151 (3)
O2—H2⋯S2^ii^	0.83 (2)	2.599 (14)	3.409 (2)	165 (4)

Symmetry codes: (i) 
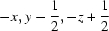
; (ii) 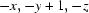.

**Fig. 1.**
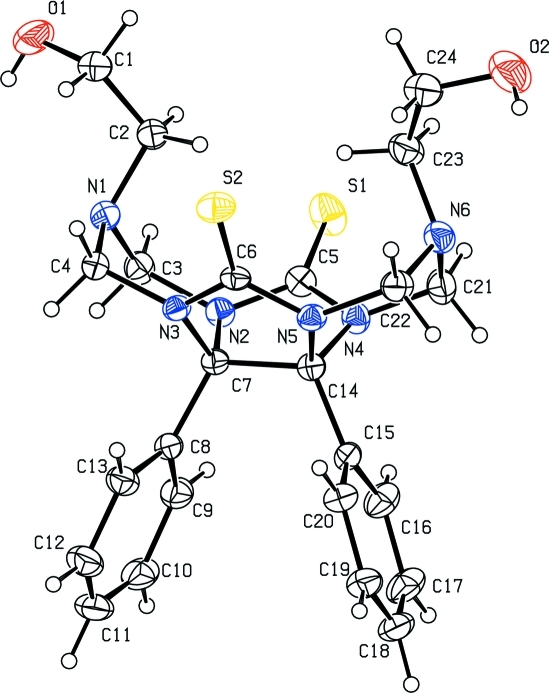
The molecular structure of the title molecule, with the atom-numbering scheme. Displacement ellipsoids are drawn at the 30% probability level.

## supplementary crystallographic information

### Comment

The rigid concave shape of glycoluril makes it a versatile building block to construct various supramolecular objects (Gao *et al.*, 2009). We report here the structure of the title thioglycoluril derivative (Fig. 1), which is a potential receptor in supramolecular chemistry.

The title compound, C_24_H_28_N_6_O_2_S_2_, is a thioglycoluril derivative. The crystal packing is stabilized by intermolecular O–H···S hydrogen bonds (Table 1).

### Experimental

The title compound was synthesized according to the procedure reported (Broan *et al.*, 1989; Li *et al.*, 2006). Crystals appropriate for X-ray data collection were obtained by slow evaporation of the dichloromethane solution at 293 K.

### Refinement

C-bound H atoms were positioned in geometrically idealized positions and constrained to ride on their parent atoms, with C—H distances in the range 0.93–0.97 Å and *U*_iso_(H) = 1.2*U*_eq_(C) or *U*_iso_(H) = 1.5*U*_eq_(C). The hydroxyl H atoms were found from the Fourier difference map and refined with the bond restraint O—H = 0.82 (2) Å, and *U*_iso_(H) = 1.5*U*_eq_(O).

### Crystal data

**Table:**

C_24_H_28_N_6_O_2_S_2_	*F*(000) = 1048
*M**_r_* = 496.64	*D*_x_ = 1.373 Mg m^−^^3^
Monoclinic, *P*2_1_/*c*	Mo *K*α radiation, λ = 0.71073 Å
Hall symbol: -P 2ybc	Cell parameters from 7811 reflections
*a* = 10.8207 (3) Å	θ = 2.6–26.8°
*b* = 11.9259 (3) Å	µ = 0.26 mm^−^^1^
*c* = 18.7222 (5) Å	*T* = 295 K
β = 95.917 (1)°	Block, colorless
*V* = 2403.16 (11) Å^3^	0.30 × 0.20 × 0.10 mm
*Z* = 4	

### Data collection

**Table:**

Bruker SMART APEX CCD area-detector diffractometer	5248 independent reflections
Radiation source: fine-focus sealed tube	3908 reflections with *I* > 2σ(*I*)
graphite	*R*_int_ = 0.048
φ and ω scans	θ_max_ = 27.0°, θ_min_ = 1.9°
Absorption correction: multi-scan (*SADABS*; Sheldrick,1996)	*h* = −13→13
*T*_min_ = 0.927, *T*_max_ = 0.975	*k* = −15→14
26638 measured reflections	*l* = −23→23

### Refinement

**Table:**

Refinement on *F*^2^	Primary atom site location: structure-invariant direct methods
Least-squares matrix: full	Secondary atom site location: difference Fourier map
*R*[*F*^2^ > 2σ(*F*^2^)] = 0.049	Hydrogen site location: inferred from neighbouring sites
*wR*(*F*^2^) = 0.141	H atoms treated by a mixture of independent and constrained refinement
*S* = 1.04	*w* = 1/[σ^2^(*F*_o_^2^) + (0.086*P*)^2^] where *P* = (*F*_o_^2^ + 2*F*_c_^2^)/3
5248 reflections	(Δ/σ)_max_ < 0.001
313 parameters	Δρ_max_ = 0.43 e Å^−^^3^
2 restraints	Δρ_min_ = −0.31 e Å^−^^3^

### Special details

**Table:**

Geometry. All e.s.d.'s (except the e.s.d. in the dihedral angle between two l.s. planes) are estimated using the full covariance matrix. The cell e.s.d.'s are taken into account individually in the estimation of e.s.d.'s in distances, angles and torsion angles; correlations between e.s.d.'s in cell parameters are only used when they are defined by crystal symmetry. An approximate (isotropic) treatment of cell e.s.d.'s is used for estimating e.s.d.'s involving l.s. planes.
Refinement. Refinement of *F*^2^ against ALL reflections. The weighted *R*-factor *wR* and goodness of fit *S* are based on *F*^2^, conventional *R*-factors *R* are based on *F*, with *F* set to zero for negative *F*^2^. The threshold expression of *F*^2^ > σ(*F*^2^) is used only for calculating *R*-factors(gt) *etc*. and is not relevant to the choice of reflections for refinement. *R*-factors based on *F*^2^ are statistically about twice as large as those based on *F*, and *R*- factors based on ALL data will be even larger.

### Fractional atomic coordinates and isotropic or equivalent isotropic displacement parameters (Å^2^)

**Table:**

	*x*	*y*	*z*	*U*_iso_*/*U*_eq_	
C1	−0.12009 (19)	0.06722 (19)	0.17347 (12)	0.0556 (6)	
H1A	−0.1247	0.1295	0.2064	0.067*	
H1B	−0.1835	0.0784	0.1336	0.067*	
C2	0.00508 (18)	0.06743 (18)	0.14581 (10)	0.0468 (5)	
H2A	0.0141	0.0001	0.1177	0.056*	
H2B	0.0113	0.1316	0.1146	0.056*	
C3	0.22092 (19)	0.02137 (15)	0.18881 (10)	0.0423 (5)	
H3A	0.2027	−0.0508	0.1663	0.051*	
H3B	0.2735	0.0084	0.2332	0.051*	
C4	0.12806 (17)	0.18506 (15)	0.23345 (9)	0.0389 (4)	
H4A	0.1770	0.1812	0.2798	0.047*	
H4B	0.0491	0.2199	0.2403	0.047*	
C5	0.27962 (17)	0.08201 (15)	0.06750 (10)	0.0376 (4)	
C6	0.14142 (15)	0.32561 (14)	0.13366 (9)	0.0322 (4)	
C7	0.31088 (15)	0.20650 (13)	0.16475 (9)	0.0302 (4)	
C8	0.41469 (15)	0.21794 (15)	0.22511 (9)	0.0350 (4)	
C9	0.51836 (17)	0.14859 (17)	0.22663 (11)	0.0459 (5)	
H9	0.5209	0.0914	0.1930	0.055*	
C10	0.61795 (18)	0.1651 (2)	0.27853 (13)	0.0579 (6)	
H10	0.6882	0.1201	0.2790	0.070*	
C11	0.6129 (2)	0.2475 (2)	0.32904 (13)	0.0617 (6)	
H11	0.6794	0.2575	0.3641	0.074*	
C12	0.5107 (2)	0.31542 (19)	0.32845 (12)	0.0566 (6)	
H12	0.5079	0.3709	0.3632	0.068*	
C13	0.41094 (18)	0.30164 (16)	0.27611 (10)	0.0428 (4)	
H13	0.3420	0.3484	0.2754	0.051*	
C14	0.32978 (15)	0.26964 (13)	0.09338 (9)	0.0318 (4)	
C15	0.45229 (15)	0.33084 (15)	0.09195 (9)	0.0354 (4)	
C16	0.55620 (18)	0.27555 (18)	0.07309 (12)	0.0510 (5)	
H16	0.5494	0.2025	0.0557	0.061*	
C17	0.67082 (19)	0.3290 (2)	0.08007 (13)	0.0599 (6)	
H17	0.7408	0.2914	0.0676	0.072*	
C18	0.68142 (19)	0.4367 (2)	0.10520 (13)	0.0577 (6)	
H18	0.7589	0.4712	0.1107	0.069*	
C19	0.5778 (2)	0.49443 (19)	0.12239 (12)	0.0523 (5)	
H19	0.5848	0.5682	0.1384	0.063*	
C20	0.46311 (17)	0.44085 (16)	0.11538 (11)	0.0441 (5)	
H20	0.3929	0.4793	0.1266	0.053*	
C21	0.28287 (19)	0.22150 (17)	−0.03467 (10)	0.0464 (5)	
H21A	0.3540	0.2611	−0.0498	0.056*	
H21B	0.2662	0.1576	−0.0663	0.056*	
C22	0.19164 (19)	0.38613 (15)	0.01067 (11)	0.0428 (5)	
H22A	0.1156	0.4296	0.0086	0.051*	
H22B	0.2573	0.4354	−0.0019	0.051*	
C23	0.0576 (2)	0.23381 (17)	−0.04220 (12)	0.0537 (6)	
H23A	0.0491	0.2073	0.0060	0.064*	
H23B	0.0610	0.1686	−0.0729	0.064*	
C24	−0.0547 (2)	0.3012 (2)	−0.06773 (14)	0.0681 (7)	
H24A	−0.1281	0.2574	−0.0610	0.082*	
H24B	−0.0563	0.3676	−0.0379	0.082*	
N1	0.10632 (14)	0.07216 (13)	0.20529 (8)	0.0414 (4)	
N2	0.28966 (13)	0.09106 (11)	0.14054 (8)	0.0342 (3)	
N3	0.19398 (12)	0.25404 (12)	0.18405 (7)	0.0319 (3)	
N4	0.31342 (14)	0.18107 (12)	0.03940 (8)	0.0373 (4)	
N5	0.22274 (13)	0.34526 (12)	0.08454 (8)	0.0333 (3)	
N6	0.17545 (15)	0.29589 (13)	−0.04166 (8)	0.0453 (4)	
O1	−0.14482 (17)	−0.03208 (18)	0.20862 (11)	0.0846 (6)	
H1	−0.087 (2)	−0.045 (3)	0.2388 (15)	0.127*	
O2	−0.0615 (2)	0.33480 (17)	−0.13943 (11)	0.0862 (6)	
H2	−0.033 (3)	0.3995 (15)	−0.1335 (19)	0.129*	
S1	0.24080 (7)	−0.03339 (4)	0.02083 (3)	0.0601 (2)	
S2	0.00120 (4)	0.38457 (5)	0.13406 (3)	0.04925 (17)	

### Atomic displacement parameters (Å^2^)

**Table:**

	*U*^11^	*U*^22^	*U*^33^	*U*^12^	*U*^13^	*U*^23^
C1	0.0446 (12)	0.0720 (15)	0.0484 (13)	−0.0162 (11)	−0.0034 (10)	0.0076 (11)
C2	0.0515 (12)	0.0500 (12)	0.0384 (11)	−0.0192 (9)	0.0022 (9)	−0.0006 (9)
C3	0.0530 (11)	0.0342 (10)	0.0391 (11)	−0.0063 (8)	0.0023 (9)	0.0039 (8)
C4	0.0369 (10)	0.0488 (11)	0.0313 (10)	−0.0088 (8)	0.0051 (8)	−0.0008 (8)
C5	0.0393 (10)	0.0346 (9)	0.0388 (11)	0.0028 (8)	0.0031 (8)	−0.0017 (8)
C6	0.0281 (8)	0.0316 (9)	0.0361 (10)	−0.0041 (7)	−0.0006 (7)	−0.0059 (7)
C7	0.0279 (8)	0.0303 (8)	0.0323 (9)	0.0002 (6)	0.0028 (7)	−0.0009 (7)
C8	0.0301 (9)	0.0389 (10)	0.0354 (10)	−0.0043 (7)	0.0008 (7)	0.0055 (8)
C9	0.0360 (10)	0.0533 (12)	0.0481 (12)	0.0027 (9)	0.0033 (9)	0.0075 (9)
C10	0.0316 (10)	0.0721 (15)	0.0688 (16)	0.0026 (10)	−0.0008 (10)	0.0249 (13)
C11	0.0438 (13)	0.0765 (16)	0.0600 (15)	−0.0198 (11)	−0.0178 (11)	0.0157 (13)
C12	0.0595 (14)	0.0585 (13)	0.0485 (13)	−0.0183 (11)	−0.0109 (10)	−0.0011 (10)
C13	0.0429 (10)	0.0417 (10)	0.0422 (11)	−0.0050 (8)	−0.0029 (8)	−0.0009 (8)
C14	0.0312 (9)	0.0308 (9)	0.0334 (9)	0.0007 (7)	0.0041 (7)	−0.0024 (7)
C15	0.0308 (9)	0.0414 (10)	0.0345 (10)	−0.0021 (7)	0.0062 (7)	0.0051 (8)
C16	0.0398 (11)	0.0517 (12)	0.0636 (14)	0.0056 (9)	0.0155 (10)	0.0069 (10)
C17	0.0328 (11)	0.0704 (15)	0.0789 (17)	0.0057 (10)	0.0174 (10)	0.0173 (13)
C18	0.0323 (10)	0.0753 (16)	0.0647 (15)	−0.0137 (10)	0.0008 (10)	0.0233 (12)
C19	0.0448 (11)	0.0550 (12)	0.0564 (13)	−0.0150 (10)	0.0016 (9)	0.0039 (10)
C20	0.0344 (10)	0.0466 (11)	0.0513 (12)	−0.0054 (8)	0.0050 (8)	−0.0008 (9)
C21	0.0574 (13)	0.0478 (11)	0.0348 (11)	−0.0032 (9)	0.0079 (9)	−0.0012 (9)
C22	0.0426 (10)	0.0369 (10)	0.0479 (12)	−0.0023 (8)	−0.0005 (9)	0.0099 (9)
C23	0.0595 (14)	0.0503 (12)	0.0481 (13)	−0.0145 (10)	−0.0098 (10)	0.0072 (10)
C24	0.0584 (15)	0.0822 (17)	0.0611 (16)	−0.0197 (13)	−0.0068 (12)	0.0089 (13)
N1	0.0435 (9)	0.0430 (9)	0.0375 (9)	−0.0118 (7)	0.0032 (7)	0.0011 (7)
N2	0.0400 (8)	0.0283 (7)	0.0341 (8)	−0.0022 (6)	0.0027 (6)	−0.0023 (6)
N3	0.0258 (7)	0.0364 (8)	0.0337 (8)	−0.0021 (6)	0.0043 (6)	−0.0027 (6)
N4	0.0446 (9)	0.0356 (8)	0.0323 (8)	−0.0011 (7)	0.0062 (7)	−0.0035 (6)
N5	0.0281 (7)	0.0341 (8)	0.0374 (8)	0.0006 (6)	0.0011 (6)	0.0034 (6)
N6	0.0508 (10)	0.0446 (9)	0.0390 (9)	−0.0074 (7)	−0.0022 (7)	0.0054 (7)
O1	0.0646 (12)	0.1027 (14)	0.0842 (14)	−0.0396 (11)	−0.0034 (9)	0.0390 (11)
O2	0.0899 (14)	0.0982 (15)	0.0647 (12)	−0.0084 (12)	−0.0198 (10)	0.0193 (11)
S1	0.0926 (5)	0.0386 (3)	0.0479 (4)	−0.0061 (3)	0.0013 (3)	−0.0121 (2)
S2	0.0294 (3)	0.0545 (3)	0.0636 (4)	0.0086 (2)	0.0036 (2)	−0.0030 (3)

### Geometric parameters (Å, °)

**Table:**

C1—O1	1.394 (3)	C12—H12	0.9300
C1—C2	1.499 (3)	C13—H13	0.9300
C1—H1A	0.9700	C14—N4	1.460 (2)
C1—H1B	0.9700	C14—N5	1.464 (2)
C2—N1	1.481 (3)	C14—C15	1.516 (2)
C2—H2A	0.9700	C15—C16	1.380 (2)
C2—H2B	0.9700	C15—C20	1.384 (3)
C3—N1	1.442 (2)	C16—C17	1.388 (3)
C3—N2	1.483 (2)	C16—H16	0.9300
C3—H3A	0.9700	C17—C18	1.369 (3)
C3—H3B	0.9700	C17—H17	0.9300
C4—N1	1.456 (2)	C18—C19	1.381 (3)
C4—N3	1.476 (2)	C18—H18	0.9300
C4—H4A	0.9700	C19—C20	1.390 (3)
C4—H4B	0.9700	C19—H19	0.9300
C5—N4	1.359 (2)	C20—H20	0.9300
C5—N2	1.365 (2)	C21—N6	1.457 (2)
C5—S1	1.6609 (19)	C21—N4	1.473 (2)
C6—N3	1.353 (2)	C21—H21A	0.9700
C6—N5	1.357 (2)	C21—H21B	0.9700
C6—S2	1.6730 (17)	C22—N6	1.454 (2)
C7—N2	1.460 (2)	C22—N5	1.472 (2)
C7—N3	1.465 (2)	C22—H22A	0.9700
C7—C8	1.515 (2)	C22—H22B	0.9700
C7—C14	1.565 (2)	C23—N6	1.474 (2)
C8—C13	1.385 (3)	C23—C24	1.494 (3)
C8—C9	1.392 (3)	C23—H23A	0.9700
C9—C10	1.389 (3)	C23—H23B	0.9700
C9—H9	0.9300	C24—O2	1.395 (3)
C10—C11	1.369 (3)	C24—H24A	0.9700
C10—H10	0.9300	C24—H24B	0.9700
C11—C12	1.370 (3)	O1—H1	0.81 (2)
C11—H11	0.9300	O2—H2	0.83 (2)
C12—C13	1.390 (3)		
			
O1—C1—C2	112.9 (2)	C16—C15—C14	120.75 (16)
O1—C1—H1A	109.0	C20—C15—C14	119.75 (15)
C2—C1—H1A	109.0	C15—C16—C17	120.0 (2)
O1—C1—H1B	109.0	C15—C16—H16	120.0
C2—C1—H1B	109.0	C17—C16—H16	120.0
H1A—C1—H1B	107.8	C18—C17—C16	120.3 (2)
N1—C2—C1	111.41 (16)	C18—C17—H17	119.8
N1—C2—H2A	109.3	C16—C17—H17	119.8
C1—C2—H2A	109.3	C17—C18—C19	120.44 (19)
N1—C2—H2B	109.3	C17—C18—H18	119.8
C1—C2—H2B	109.3	C19—C18—H18	119.8
H2A—C2—H2B	108.0	C18—C19—C20	119.1 (2)
N1—C3—N2	113.05 (14)	C18—C19—H19	120.4
N1—C3—H3A	109.0	C20—C19—H19	120.4
N2—C3—H3A	109.0	C15—C20—C19	120.72 (19)
N1—C3—H3B	109.0	C15—C20—H20	119.6
N2—C3—H3B	109.0	C19—C20—H20	119.6
H3A—C3—H3B	107.8	N6—C21—N4	112.54 (15)
N1—C4—N3	111.04 (13)	N6—C21—H21A	109.1
N1—C4—H4A	109.4	N4—C21—H21A	109.1
N3—C4—H4A	109.4	N6—C21—H21B	109.1
N1—C4—H4B	109.4	N4—C21—H21B	109.1
N3—C4—H4B	109.4	H21A—C21—H21B	107.8
H4A—C4—H4B	108.0	N6—C22—N5	112.82 (14)
N4—C5—N2	108.81 (15)	N6—C22—H22A	109.0
N4—C5—S1	125.48 (14)	N5—C22—H22A	109.0
N2—C5—S1	125.61 (14)	N6—C22—H22B	109.0
N3—C6—N5	109.03 (14)	N5—C22—H22B	109.0
N3—C6—S2	125.32 (13)	H22A—C22—H22B	107.8
N5—C6—S2	125.60 (13)	N6—C23—C24	113.98 (18)
N2—C7—N3	109.23 (13)	N6—C23—H23A	108.8
N2—C7—C8	113.40 (14)	C24—C23—H23A	108.8
N3—C7—C8	111.95 (13)	N6—C23—H23B	108.8
N2—C7—C14	102.63 (13)	C24—C23—H23B	108.8
N3—C7—C14	102.50 (13)	H23A—C23—H23B	107.7
C8—C7—C14	116.19 (13)	O2—C24—C23	115.0 (2)
C13—C8—C9	119.68 (17)	O2—C24—H24A	108.5
C13—C8—C7	120.47 (16)	C23—C24—H24A	108.5
C9—C8—C7	119.72 (16)	O2—C24—H24B	108.5
C10—C9—C8	119.7 (2)	C23—C24—H24B	108.5
C10—C9—H9	120.1	H24A—C24—H24B	107.5
C8—C9—H9	120.1	C3—N1—C4	110.83 (14)
C11—C10—C9	120.1 (2)	C3—N1—C2	114.15 (15)
C11—C10—H10	119.9	C4—N1—C2	112.76 (15)
C9—C10—H10	119.9	C5—N2—C7	112.29 (14)
C10—C11—C12	120.6 (2)	C5—N2—C3	124.97 (15)
C10—C11—H11	119.7	C7—N2—C3	114.32 (13)
C12—C11—H11	119.7	C6—N3—C7	112.56 (13)
C11—C12—C13	120.2 (2)	C6—N3—C4	126.37 (14)
C11—C12—H12	119.9	C7—N3—C4	115.11 (13)
C13—C12—H12	119.9	C5—N4—C14	112.29 (14)
C8—C13—C12	119.69 (19)	C5—N4—C21	127.29 (15)
C8—C13—H13	120.2	C14—N4—C21	114.47 (14)
C12—C13—H13	120.2	C6—N5—C14	112.19 (14)
N4—C14—N5	109.23 (14)	C6—N5—C22	126.32 (14)
N4—C14—C15	112.33 (13)	C14—N5—C22	114.32 (14)
N5—C14—C15	112.61 (13)	C22—N6—C21	110.52 (15)
N4—C14—C7	103.04 (13)	C22—N6—C23	114.88 (16)
N5—C14—C7	102.79 (12)	C21—N6—C23	112.11 (16)
C15—C14—C7	115.99 (14)	C1—O1—H1	108 (3)
C16—C15—C20	119.29 (17)	C24—O2—H2	99 (3)
			
O1—C1—C2—N1	−69.4 (2)	C8—C7—N2—C5	−133.09 (15)
N2—C7—C8—C13	−148.92 (15)	C14—C7—N2—C5	−6.95 (17)
N3—C7—C8—C13	−24.8 (2)	N3—C7—N2—C3	−48.59 (19)
C14—C7—C8—C13	92.50 (19)	C8—C7—N2—C3	77.02 (18)
N2—C7—C8—C9	35.3 (2)	C14—C7—N2—C3	−156.84 (14)
N3—C7—C8—C9	159.47 (15)	N1—C3—N2—C5	−93.2 (2)
C14—C7—C8—C9	−83.3 (2)	N1—C3—N2—C7	52.3 (2)
C13—C8—C9—C10	−1.1 (3)	N5—C6—N3—C7	−9.10 (19)
C7—C8—C9—C10	174.74 (17)	S2—C6—N3—C7	173.37 (12)
C8—C9—C10—C11	1.6 (3)	N5—C6—N3—C4	−160.35 (15)
C9—C10—C11—C12	−0.9 (3)	S2—C6—N3—C4	22.1 (2)
C10—C11—C12—C13	−0.4 (3)	N2—C7—N3—C6	−103.97 (15)
C9—C8—C13—C12	−0.2 (3)	C8—C7—N3—C6	129.58 (15)
C7—C8—C13—C12	−175.92 (17)	C14—C7—N3—C6	4.36 (17)
C11—C12—C13—C8	0.9 (3)	N2—C7—N3—C4	50.70 (18)
N2—C7—C14—N4	1.33 (15)	C8—C7—N3—C4	−75.75 (17)
N3—C7—C14—N4	−111.96 (13)	C14—C7—N3—C4	159.04 (13)
C8—C7—C14—N4	125.65 (15)	N1—C4—N3—C6	95.90 (19)
N2—C7—C14—N5	114.86 (13)	N1—C4—N3—C7	−54.72 (19)
N3—C7—C14—N5	1.57 (15)	N2—C5—N4—C14	−9.2 (2)
C8—C7—C14—N5	−120.81 (15)	S1—C5—N4—C14	174.18 (13)
N2—C7—C14—C15	−121.82 (15)	N2—C5—N4—C21	−160.26 (16)
N3—C7—C14—C15	124.89 (14)	S1—C5—N4—C21	23.2 (3)
C8—C7—C14—C15	2.5 (2)	N5—C14—N4—C5	−104.12 (16)
N4—C14—C15—C16	−32.1 (2)	C15—C14—N4—C5	130.19 (16)
N5—C14—C15—C16	−155.98 (17)	C7—C14—N4—C5	4.64 (18)
C7—C14—C15—C16	86.0 (2)	N5—C14—N4—C21	50.83 (18)
N4—C14—C15—C20	153.17 (17)	C15—C14—N4—C21	−74.86 (18)
N5—C14—C15—C20	29.3 (2)	C7—C14—N4—C21	159.59 (14)
C7—C14—C15—C20	−88.7 (2)	N6—C21—N4—C5	96.9 (2)
C20—C15—C16—C17	2.2 (3)	N6—C21—N4—C14	−53.6 (2)
C14—C15—C16—C17	−172.49 (19)	N3—C6—N5—C14	10.25 (19)
C15—C16—C17—C18	−0.4 (3)	S2—C6—N5—C14	−172.23 (12)
C16—C17—C18—C19	−1.5 (4)	N3—C6—N5—C22	158.67 (15)
C17—C18—C19—C20	1.5 (3)	S2—C6—N5—C22	−23.8 (2)
C16—C15—C20—C19	−2.2 (3)	N4—C14—N5—C6	101.81 (16)
C14—C15—C20—C19	172.53 (18)	C15—C14—N5—C6	−132.65 (15)
C18—C19—C20—C15	0.4 (3)	C7—C14—N5—C6	−7.11 (17)
N6—C23—C24—O2	65.1 (3)	N4—C14—N5—C22	−50.61 (18)
N2—C3—N1—C4	−53.7 (2)	C15—C14—N5—C22	74.93 (18)
N2—C3—N1—C2	74.92 (19)	C7—C14—N5—C22	−159.53 (13)
N3—C4—N1—C3	54.3 (2)	N6—C22—N5—C6	−94.4 (2)
N3—C4—N1—C2	−75.12 (18)	N6—C22—N5—C14	53.41 (19)
C1—C2—N1—C3	152.21 (17)	N5—C22—N6—C21	−52.6 (2)
C1—C2—N1—C4	−80.1 (2)	N5—C22—N6—C23	75.5 (2)
N4—C5—N2—C7	10.2 (2)	N4—C21—N6—C22	52.5 (2)
S1—C5—N2—C7	−173.19 (13)	N4—C21—N6—C23	−77.0 (2)
N4—C5—N2—C3	156.33 (16)	C24—C23—N6—C22	67.6 (2)
S1—C5—N2—C3	−27.1 (3)	C24—C23—N6—C21	−165.17 (18)
N3—C7—N2—C5	101.30 (16)		

### Hydrogen-bond geometry (Å, °)

**Table:**

*D*—H···*A*	*D*—H	H···*A*	*D*···*A*	*D*—H···*A*
O1—H1···S2^i^	0.81 (2)	2.61 (2)	3.337 (2)	151 (3)
O2—H2···S2^ii^	0.83 (2)	2.60 (1)	3.409 (2)	165 (4)

Symmetry codes: (i) −*x*, *y*−1/2, −*z*+1/2; (ii) −*x*, −*y*+1, −*z*.
